# Efficacy of Lactobacillus Reuteri DSM 17938 for the Treatment of Acute Gastroenteritis in Children: Protocol of a Randomized Controlled Trial

**DOI:** 10.2196/resprot.7924

**Published:** 2017-08-23

**Authors:** Henryk Szymański, Hania Szajewska

**Affiliations:** ^1^ Department of Pediatrics St Hedwig of Silesia Hospital Trzebnica Poland; ^2^ Department of Pediatrics The Medical University of Warsaw Warsaw Poland

**Keywords:** probiotics, diarrhea, infants, RCT

## Abstract

**Background:**

Acute gastroenteritis (AGE) is one of the most common diseases among children. Oral rehydration therapy is the key treatment. However, despite proven efficacy, it remains underused. This is because oral rehydration solution neither reduces the frequency of bowel movements and fluid loss nor shortens the duration of illness. Hence, there is interest in adjunctive treatments. According to the 2014 guidelines developed by the European Society for Paediatric Gastroenterology, Hepatology and Nutrition, the use of the following probiotics may be considered in the management of children with AGE in addition to rehydration therapy: *Lactobacillus rhamnosus* GG (low quality of evidence; strong recommendation) and *Saccharomyces boulardii* (low quality of evidence; strong recommendation). Less compelling evidence is available for *Lactobacillus reuteri* DSM 17938 (very low quality of evidence; weak recommendation).

**Objective:**

Considering that evidence on *L reuteri* remains limited, the goal of the study is to assess the effectiveness of *L reuteri* DSM 17938 in the treatment of AGE in children. Children vaccinated and not vaccinated against rotavirus will be evaluated separately.

**Methods:**

This will be a double-blind, placebo-controlled, randomized trial. Children between 1 and 60 months of age with AGE, defined as a change in stool consistency to loose or liquid form (according to the Bristol Stool Form scale or Amsterdam Stool Form scale) and/or an increase in the frequency of evacuations (typically ≥3 in 24 h) lasting for no longer than 5 days, will be recruited. A total of 72 children will receive either *L reuteri* DSM 17938 at a dose of 2×10^8^colony-forming units twice daily or matching placebo for 5 consecutive days. A similar sample size for rotavirus vaccinated and nonvaccinated children is planned. The primary outcome measure is the duration of diarrhea. Two separate studies and reports for rotavirus vaccinated and nonvaccinated children are planned.

**Results:**

The recruitment started in January 2017 and is planned to be finalized in June 2018 for rotavirus nonvaccinated children. The recruitment of rotavirus-vaccinated children may be slower due to a relatively low coverage rate in Poland. Data analysis and submission to a peer-reviewed journal is expected within 3 months after completion of the study.

**Conclusion:**

This study will add to current knowledge on the efficacy of *L reuteri* DSM 17938 for the management of AGE.

**Trial registration:**

ClinicalTrials.gov NCT02989350; https://clinicaltrials.gov/ct2/show/NCT02989350 (Archived by WebCite at http://www.webcitation.org/6slOFkyTH)

## Introduction

Acute gastroenteritis (AGE) is one of the most common diseases among children. Generally, this is a self-limited illness lasting 5 to 7 days, and thus, the main aim of treatment is to prevent dehydration, metabolic acidosis, and electrolyte disturbances. In the vast majority of cases of AGE with mild or moderate dehydration, this can be achieved with oral rehydration solutions. Despite the proven efficacy of oral rehydration, it remains underused [1]. The main reason for this is that an oral rehydration solution neither reduces the frequency of bowel movements and fluid loss nor shortens the duration of illness, which decreases its acceptance and prompts interest in adjunctive treatments.

In 2014, the European Society for Paediatric Gastroenterology, Hepatology and Nutrition (ESPGHAN) provided recommendations for the use of probiotics for the treatment of AGE in previously healthy infants and children based on a systematic review. The use of the following probiotics may be considered in the management of children with AGE in addition to rehydration therapy: *Lactobacillus rhamnosus* GG (low quality of evidence; strong recommendation) and *Saccharomyces boulardii* (low quality of evidence; strong recommendation). Less compelling evidence is available for *Lactobacillus reuteri* DSM 17938 (very low quality of evidence; weak recommendation) [2,3].

*L reuteri* is named after Gerhard Reuter, a German microbiologist [4]. Among a number of *L reuteri* strains, *L reuteri* DSM 17938 is probably the best studied. *L reuteri* DSM 17938 is the daughter strain of *L reuteri* ATCC 55730. The latter was originally isolated from the breast milk of a Peruvian mother, and it may be present in normal humans on the mucosa of the gastric corpus and antrum, duodenum, and ileum [4,5]. As *L reuteri* ATCC 55730 was found to carry potentially transferable resistance traits for tetracycline and lincomycin, it was replaced by *L reuteri* DSM 17938, a strain without unwanted plasmid-borne resistance [6]. Like other *L reuteri* strains, *L reuteri* DSM 17938 has several different types of mechanisms of action. One of the best-documented mechanisms is its antimicrobial activity. *L reuteri* strains produce reuterin, a broad-spectrum antibacterial substance [7,8], which is capable of inhibiting the growth of a wide spectrum of microorganisms such as Gram-positive or negative bacteria, yeast, fungi, or parasites [9]. *L reuteri* strains may also regulate immune responses [10-12].

The clinical efficacy of *L reuteri* DSM 17938 has been studied in a number of clinical trials, including studies aimed at the assessment of *L reuteri* DSM 17938 in the management of AGE.

A 2012 study by Francavilla et al [13] was carried out in 74 children with acute diarrhea randomized to receive *L reuteri* DSM 17938 (at a dose of 4×10^8^colony-forming units [CFU]) or placebo for 7 days. Compared with the placebo group, in the *L reuteri* group there was a significant reduction in the duration of diarrhea (3.3 [SD 2.1] vs 2.1 [SD 1.7] days, respectively; *P*<.03), the risk of watery diarrhea on day 2 (81% vs 55%, respectively, *P*<.02) and on day 3, 73% vs 46%, respectively, *P*<.03), and the risk of relapse of diarrhea (42% vs 15%; respectively, *P*<.03). There was not a significant difference in hospital stay between the groups.

A 2014 multicenter, randomized, single-blind clinical trial by Dinleyici et al [14] was performed in 127 hospitalized children with AGE lasting 12 to 72 hours. Conventional therapy plus the administration of *L reuteri* DSM 17938 at a dose 1×10^8^CFU for 5 days compared with conventional therapy alone (control group) reduced the duration of diarrhea (70.7 [SD 26.1] vs 103.8 [SD 28.47] hours, respectively; *P*<.001), increased the number of diarrhea-free children after 24 and 48 hours (50% vs 5%, respectively, *P*<.001) and 72 hours (69% vs 11%, respectively, *P*<.001), and reduced the hospital stay (4.3 [SD 1.3] days vs 5.46 [SD 1.77] days, respectively, *P*<.001).

A 2015 trial by the same authors performed in 64 outpatient children with acute infectious diarrhea found that compared with oral rehydration therapy alone (control group), the additional administration of *L reuteri* at a dose 1×10^8^CFU for 5 days significantly reduced the duration of diarrhea (74.3 [SD 15.3] hours vs 60.4 [SD 24.5] hours, respectively, *P*<.05). The percentage of children with diarrhea after 48 hours was lower in the *L reuteri* group than the control group (13/29 vs 27/31, respectively; relative risk [RR] 0.51, 95% CI 0.34 to 0.79, *P*<.01). From the 72nd hour of the intervention onward, there was no difference between the 2 groups in the percentage of children with diarrhea [15].

A 2015 meta-analysis of these 3 randomized controlled trials (n=256) found that compared with placebo or no treatment, *L reuteri* DSM 17938 administration significantly reduced the duration of diarrhea (mean difference [MD] –24.82 hours, 95% CI –38.8 to –10.8) and increased the chance of cure on day 1 (RR 11.3, 95% CI 2.2 to 59) and day 2 (RR 4.54, 95% CI 2.0 to 10.2) [16]. However, heterogeneity and wide confidence intervals call for caution in interpreting the results. To the best of our knowledge, there are no data on the use of probiotics use in rotavirus-vaccinated children with AGE.

More studies are needed to establish the efficacy of *L reuteri* DSM 17938 in the management of AGE in children. We aim to conduct a well-designed study with sufficient power, an adequate follow-up period, and relevant clinical end points. The study will be led by a team experienced in performing clinical trials in children with AGE [17,18] and follow the same protocol as one of our recent trials on AGE [19].

The objective of this study is to assess the effectiveness of *L reuteri* DSM 17938 in the treatment of AGE in children. Children vaccinated and not vaccinated against rotavirus will be evaluated separately.

## Methods

### Study Design

This study is designed as a randomized, double-blinded, placebo-controlled trial with 1:1 allocation, and it is described in more detail in the subsequent sections. The trial was registered at ClinicalTrials.gov [NCT02989350], and any important changes in the protocol will be implemented there.

### Ethics and Dissemination

The Ethical Committee of the Lower Silesia Medical Chamber issued approval for the study before recruitment commenced. Verbal and written information regarding informed consent will be presented to the caregivers. Any modifications to the protocol that may affect the conduct of the study will be presented to the Ethical Committee. The full protocol will be available freely due to open-access publication. The findings of this randomized controlled trial will be submitted to a peer-reviewed journal. Abstracts will be submitted to relevant national and international conferences. The need for separate reporting of data on rotavirus-vaccinated and nonvaccinated populations is foreseen. The Ethics Committee did not require auditing for this study.

### Setting and Participants

The recruitment will take place in the emergency room and the Department of Paediatrics of the St Hedwig of Silesia Hospital in Trzebnica, Poland. However, inclusion of outpatients and involvement of other recruiting sites are under consideration provided the study staff is experienced, adequately trained, and competent in conducting clinical trials. The start of the recruitment is planned in January 2017, and it should be completed within the following 2 years. Participants will be randomized after their first visit to the emergency room or after admission to the hospital. Caregivers will receive oral and written information on the study. Written informed consent will by obtained by physicians involved in the study. See Textbox 1 for selection criteria.

### Interventions

The intervention under investigation is *L reuteri* DSM 17938 *.* The placebo drops consist of a mixture of pharmaceutical grade medium chain triglycerides and sunflower oil together with pharmaceutical grade silicon dioxide to give the product the correct rheological properties. The formulation is identical to the active product but without  *L reuteri* DSM 17938. In our trial, we choose to use a placebo for a comparator, as it is widely regarded as the gold standard for testing the efficacy of new treatments [20,21].

The study products (*L reuteri* DSM 17938 and placebo) will be manufactured and supplied by BioGaia as bottle with drops, free of charge. The manufacturer will have no role in the conception, protocol development, design, or conduct of the study or in the analysis or interpretation of the data.

The dose of *L reuteri* DSM 17938 will be 2×10^8^CFU. Both *L reuteri* DSM 17938 and placebo will be taken orally, 5 drops twice daily for a consecutive 5 days. Caregivers will be instructed to administer the study products at the same time of the day. If needed, discontinuation or modification of the treatment may be considered at the discretion of the physician.

Table 1 presents a timetable of activities planned during the study. For initial rehydration, all children will be treated according to the 2014 ESPGHAN recommendations (fast oral rehydration over 3 to 4 hours by mouth or via nasogastric tube with the recommended hypotonic solution) [3]. After all signs of dehydration have disappeared, oral rehydration solution will be given for ongoing losses until the diarrhea stops. Rapid reintroduction of the previous diet after successful rehydration will be recommended.

**Selection criteria.**

Inclusion criteria:

- Acute gastroenteritis defined as a change in stool consistency to loose or liquid form (according to the Bristol Stool Form scale or in the case of infants, the Amsterdam Stool Form scale) and/or an increase in the frequency of evacuations (typically ≥3 in 24 hours) lasting for no longer than 5 days
- Aged older than 1 month and younger than 60 months
- Caregiver must provide written informed consent

Exclusion criteria:

- Use of antibiotics within 2 weeks prior to enrollment
- Use of gelatine tannate, diosmectite, probiotics, racecadotril, or zinc (including zinc-containing oral rehydration solution) within 1 week prior to enrollment (a single dose is allowed)
- Breastfeeding (>50%).
- Chronic diarrheal gastrointestinal disease (eg, inflammatory bowel disease, cystic fibrosis, celiac disease, food allergy)
- Immunodeficiency
- Malnutrition (weight/height/length under 3rd percentile) (World Health Organization Child Growth Standards will be used) [20]

**Table 1:** Timetable of activities planned during the study.

Activities	Days							
	1	2	3	4	5	6	7	8
Inclusion/exclusion criteria	x							
Informed consent	x							
Enrollment	x							
Randomization	x							
Stool sample taken	x							
Intervention	x	x	x	x	x			
Follow-up						x	x	x
Return of diary								x
Concomitant medication	x	x	x	x	x	x	x	x
Adverse events	x	x	x	x	x	x	x	x

After rechecking the inclusion and exclusion criteria, participants will be assigned into 1 of 2 groups (experimental or control). Caregivers will receive a diary of symptoms to record the number of stools and stool consistency during the intervention (including recording of the timing of stools); Bristol Stool Form (BSF) and Amsterdam Stool Form (ASF) scales will be provided. Additionally, caregivers will be asked to write down any adverse events and concomitant medication during the intervention period. In line with current ESPGHAN guidelines [3], children presenting with AGE do not require routine etiological investigation; however, before the first dose of study products, a stool sample will be taken to determine the rotavirus etiology of the diarrhea with a standard immunoassay method. More specific microbiological investigations with standard stool cultures will be performed only if needed [3]. At any time, caregivers will have the right to withdraw the participating child from the study; they will be not obliged to give reasons for this decision, and there will be no effect on subsequent physician and/or institutional medical care.

### Concomitant Medications

The concomitant administration of any other medication, including antipyretics and antiemetics, will be at the discretion of the physician to provide adequate care. However, it is recommended that no unnecessary concomitant medication be used. In particular, the use of antibiotics, diosmectite, probiotics, or racecadotril (all included in the exclusion criteria) should be avoided.

### Follow-Up

All study participants will be followed up for the duration of the intervention (5 days) and then for an additional 3 days or until the cessation of diarrhea.

### Outcomes

The primary outcome will be the duration of diarrhea, defined as the time until the normalization of stool consistency according to the BSF or ASF scale (in BSF scale, numbers 1, 2, 3, 4, and 5; in ASF scale, letters B or C) or the time until the normalization of the number of stools (compared with the period before the onset of diarrhea) and the presence of normal stools for 48 hours.

Secondary outcomes will include:

- Need for intravenous rehydration
- Duration of intravenous rehydration
- Need for hospitalization of outpatients
- Number of watery stools per day
- Vomiting
- Recurrence of diarrhea (48 hours after intervention)
- Severity of diarrhea according to Vesikari scale [22]
- Use of concomitant medications
- Adverse events

### Randomization

A computer-generated randomization list will be prepared by a person with no clinical involvement in the trial using a computer program (StatsDirect, StatsDirect Ltd.) with an allocation ratio of 1:1 and a block of 6. The randomization lists will be stratified according to rotavirus vaccination status (nonvaccinated and vaccinated; the latter will be defined as receiving at least 1 dose of rotavirus vaccine).

### Allocation Concealment

The allocation sequence will be concealed from the researchers enrolling and assessing participants in sequentially numbered, white, opaque, sealed and stapled envelopes which will be opened only after getting informed consent and registering the basic demographic data to case report form (CRF). Consecutive randomization numbers will be given to participants at enrollment. The study product will be packaged as product A or product B and will be given according to the randomization list.

### Blinding

The study products (active and placebo) will be packaged in identical bottles. Contents will look and taste the same. Researchers, caregivers, outcome assessors, and a person responsible for the statistical analysis will be blinded to the intervention until the completion of the study. The information on intervention assignments will be stored in a sealed envelope in a safe place in the administrative part of the department. The personal information about potential and enrolled participants will be stored in a locker within the study site, accessible to the involved researchers only.

### Compliance

The caregivers will be asked to bring the remaining study product and diary to the study site at the end of the intervention period. Compliance with the study protocol will be checked by measuring the volume left unused. Based on previously published trials, it seems appropriate to consider those participants receiving <75% of the recommended doses as noncompliant.

### Power Calculation

The primary outcome of the study is the duration of diarrhea. Based on available data in the literature, the average duration of gastroenteritis in children is 5 to 7 days [3]. We assume that a clinically significant difference in the effectiveness of *L reuteri* DSM 17938 versus placebo will shorten the duration of symptoms by 24 hours (±24 hours). To detect such a difference in the duration of diarrhea between the study groups with a power of 90% and alpha of 0.01, a sample of 60 children is needed. Assuming approximately 20% loss to follow-up, we aim to recruit a total of 72 children for this study. A similar sample size for rotavirus vaccinated and nonvaccinated children is planned. Sample size calculations were performed with StatsDirect version 2.3.8 (StatsDirect Ltd).

At the Department of Paediatrics of St Hedwig of Silesia Hospital, there are 300 admissions of children with diarrhea per year and the same number of such patients who present to the emergency room. Assuming that 20% of these children will be eligible for the study, we will achieve adequate participant enrollment to reach the target sample size during 1 year of recruiting for rotavirus nonvaccinated children. Recruitment of rotavirus-vaccinated children may be slower (up to 2 years). In Poland, rotavirus vaccination has been recommended since 2006; however, vaccine reimbursement is not available. Hence, currently, the rotavirus vaccination coverage rate is low (12.7% [23] to 30% [24]), likely due to the high vaccine cost.

### Data Collection and Management

All study participants will be assigned a study identification number. CRFs will be completed on paper forms. Data will then be entered and stored in a password-protected electronic database. The original paper copies of CRFs and all study data will be stored in a locker within the study site, accessible to the involved researchers only.

### Statistical Analysis

All analysis will be conducted on an intention-to-treat basis, including all patients in the groups to which they are randomized for whom outcomes are available (including drop-outs and withdrawals). Descriptive statistics will be used to summarize baseline characteristics. The Student *t* test will be used to compare mean values of continuous variables for approximating a normal distribution. For nonnormally distributed variables, the Mann-Whitney U test will be used. The chi-square or Fisher exact test will be used, when appropriate, to compare percentages. For continuous outcomes, differences in means or differences in medians (depending on the distribution of the data), and for dichotomous outcomes, the RR and number needed to treat, all with a 95% CI, will be calculated. The difference between study groups will be considered significant when the *P* value is <.05, when the 95% CI for RR does not include 1.0, or when the 95% CI for MD does not include 0. All statistical tests will be 2-tailed and performed at the 5% level of significance. Two independent reports (rotavirus-vaccinated and nonvaccinated children) are planned.

### Monitoring

The study will be carried out in accordance with the approved protocol. *L reuteri* DSM 17938 is being safely used worldwide for a number of indications, and the US Food and Drug Administration applied to it the Generally Recognized as Safe status [25]. Still, an independent Data and Safety Monitoring Board (DSMB) will be set up prior to the start of the study. The DSMB will review data after recruitment of 25%, 50%, and 75% of subjects to review the study progress and all adverse events.

### Harms

Although the occurrence of adverse events as a result of participation in the current trial is not expected, data on adverse events will be collected. All serious adverse events will be immediately reported to the study coordinators who will be responsible for notifying the Ethics Committee, all participating investigators, and the manufacturer of the study products.

## Results

The recruitment started in January 2017 and is planned to be finalized in June 2018 for rotavirus nonvaccinated children. The recruitment of rotavirus-vaccinated children may be slower due to a relatively low coverage rate in Poland. Data analysis and submission to a peer-reviewed journal is expected within 3 months after completion of the study.

## Discussion

### Summary

This study will add to the current knowledge on the efficacy of *L reuteri* DSM 17938 for the management of AGE. Children vaccinated and not vaccinated against rotavirus will be evaluated separately.

### Strengths and Limitations of This Study

A strength of the study is its study design (randomized controlled trial), which is the most robust methodology to assess the effectiveness of therapeutic interventions. The findings of this randomized controlled trial, whether positive or negative, will contribute to the formulation of further recommendations on the use of *L reuteri* DSM 17938 for the treatment of AGE in children. A limitation is that stool volume, one of the objective ways of assessing the efficacy of antidiarrheal drugs, will not be assessed. In addition, the recruitment of rotavirus-vaccinated children may be slow due to a relatively low coverage rate in Poland, likely due to the high vaccine cost.

## Appendix

Multimedia Appendix 1 CONSORT-EHEALTH checklist (v1.6.1).

## Abbreviations

AGE: acute gastroenteritis
ASF: Amsterdam Stool Form
BSF: Bristol Stool Form
CFU: colony forming units
CRF: case report form
DSMB: Data Safety and Monitoring Board
ESPHGAN: European Society of Pediatric Gastroenterology, Hepatology, and Nutrition
MD: mean difference
RR: relative risk
